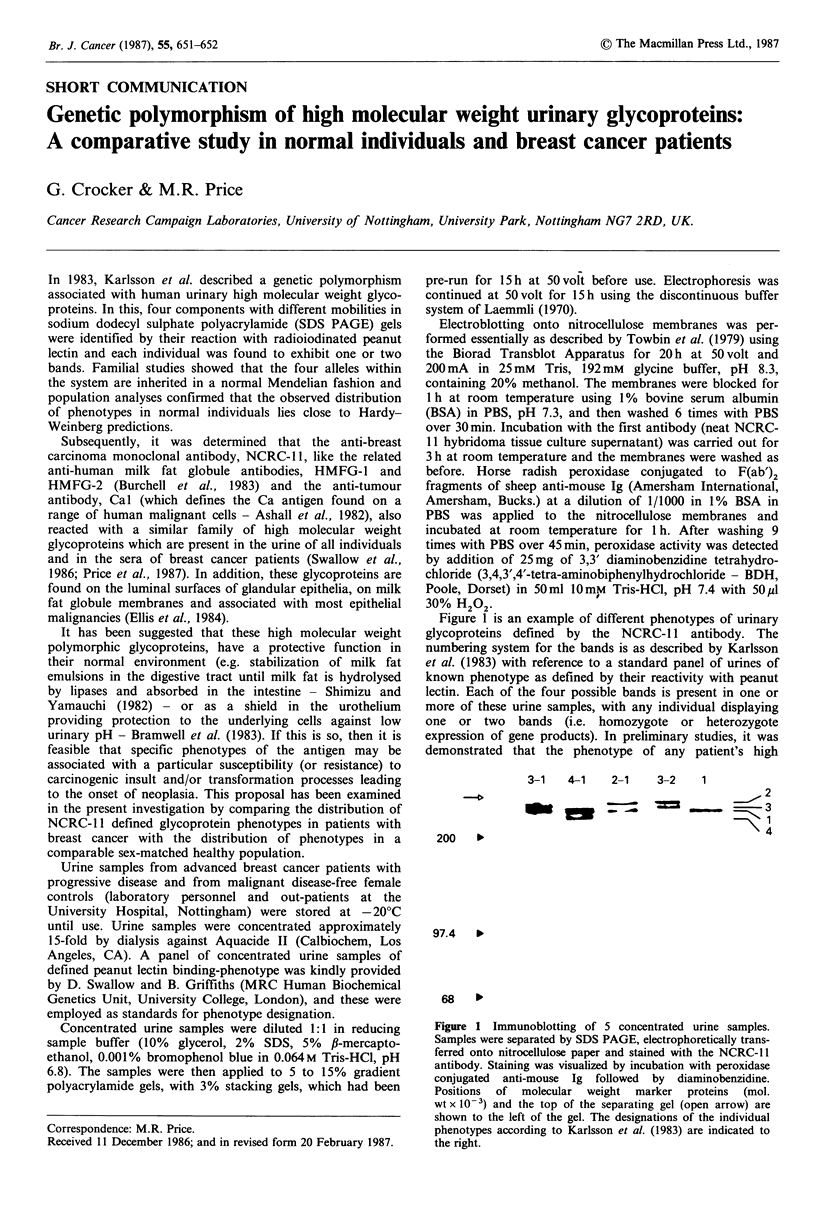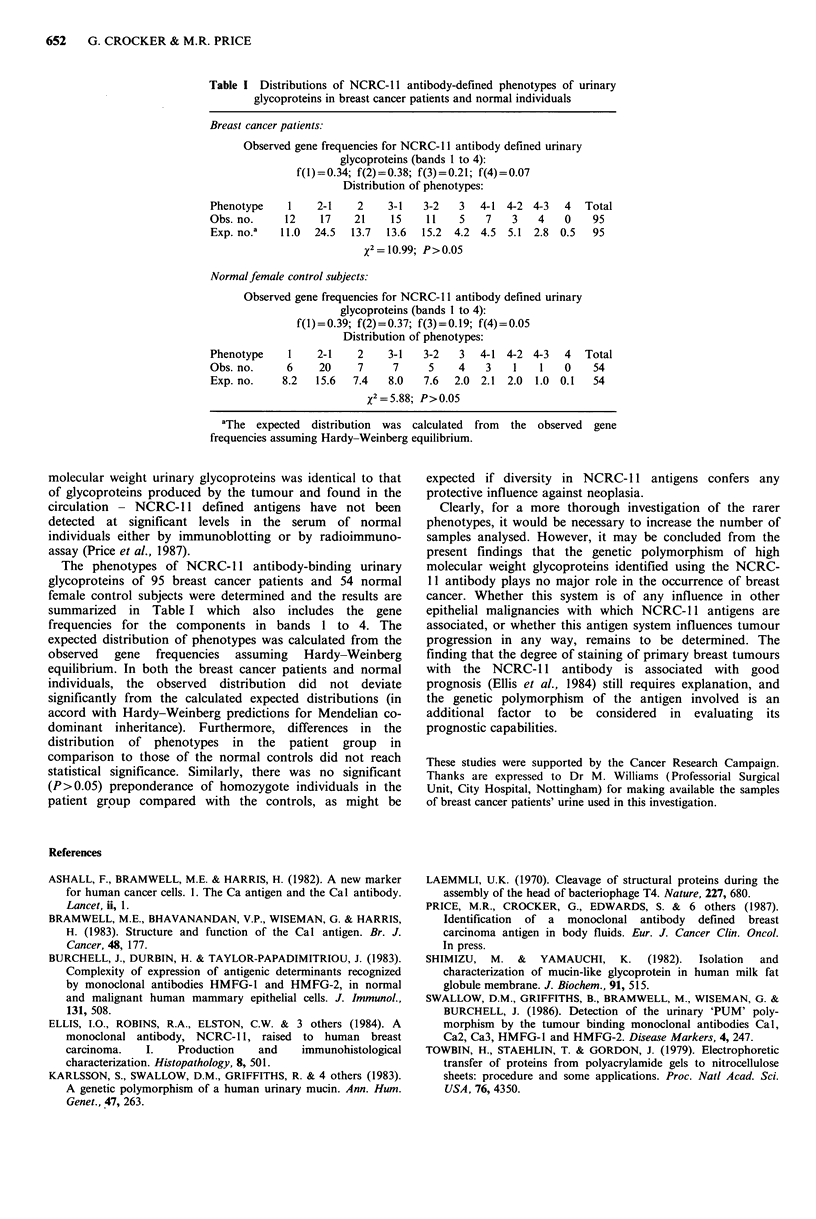# Genetic polymorphism of high molecular weight urinary glycoproteins: a comparative study in normal individuals and breast cancer patients.

**DOI:** 10.1038/bjc.1987.132

**Published:** 1987-06

**Authors:** G. Crocker, M. R. Price


*Br. J. Cancer* (1987), **55**, 651–652 © The Macmillan Press Ltd., 1987

SHORT COMMUNICATION

### Genetic polymorphism of high molecular weight urinary glycoproteins: A comparative study in normal individuals and breast cancer patients

G. Crocker & M.R. Price

*Cancer Research Campaign Laboratories, University of Nottingham, University Park, Nottingham NG7 2RD, UK.*

In 1983, Karlsson *et al.* described a genetic polymorphism associated with human urinary high molecular weight glycoproteins. In this, four components with different mobilities in sodium dodecyl sulphate polyacrylamide (SDS PAGE) gels were identified by their reaction with radioiodinated peanut lectin and each individual was found to exhibit one or two bands. Familial studies showed that the four alleles within the system are inherited in a normal Mendelian fashion and population analyses confirmed that the observed distribution of phenotypes in normal individuals lies close to Hardy–Weinberg predictions.

Subsequently, it was determined that the anti-breast carcinoma monoclonal antibody, NCRC-11, like the related anti-human milk fat globule antibodies, HMFG-1 and HMFG-2 (Burchell *et al.,* 1983) and the anti-tumour antibody, Ca1 (which defines the Ca antigen found on a range of human malignant cells – Ashall *et al.,* 1982), also reacted with a similar family of high molecular weight glycoproteins which are present in the urine of all individuals and in the sera of breast cancer patients (Swallow *et al.,* 1986; Price *et al.,* 1987). In addition, these glycoproteins are found on the luminal surfaces of glandular epithelia, on milk fat globule membranes and associated with most epithelial malignancies (Ellis *et al.,* 1984).

It has been suggested that these high molecular weight polymorphic glycoproteins, have a protective function in their normal environment (e.g. stabilization of milk fat emulsions in the digestive tract until milk fat is hydrolysed by lipases and absorbed in the intestine – Shimizu and Yamauchi (1982) – or as a shield in the urothelium providing protection to the underlying cells against low urinary pH – Bramwell *et al.* (1983). If this is so, then it is feasible that specific phenotypes of the antigen may be associated with a particular susceptibility (or resistance) to carcinogenic insult and/or transformation processes leading to the onset of neoplasia. This proposal has been examined in the present investigation by comparing the distribution of NCRC-11 defined glycoprotein phenotypes in patients with breast cancer with the distribution of phenotypes in a comparable sex-matched healthy population.

Urine samples from advanced breast cancer patients with progressive disease and from malignant disease-free female controls (laboratory personnel and out-patients at the University Hospital, Nottingham) were stored at −20°C until use. Urine samples were concentrated approximately 15-fold by dialysis against Aquacide II (Calbiochem, Los Angeles, CA). A panel of concentrated urine samples of defined peanut lectin binding-phenotype was kindly provided by D. Swallow and B. Griffiths (MRC Human Biochemical Genetics Unit, University College, London), and these were employed as standards for phenotype designation.

Concentrated urine samples were diluted 1:1 in reducing sample buffer (10% glycerol, 2% SDS, 5% $\beta$-mercapto-ethanol, 0.001% bromophenol blue in 0.064 M Tris-HCl, pH 6.8). The samples were then applied to 5 to 15% gradient polyacrylamide gels, with 3% stacking gels, which had been pre-run for 15 h at 50 volt before use. Electrophoresis was continued at 50 volt for 15 h using the discontinuous buffer system of Laemmli (1970).

Electroblotting onto nitrocellulose membranes was performed essentially as described by Towbin *et al.* (1979) using the Biorad Transblot Apparatus for 20 h at 50 volt and 200 mA in 25 mM Tris, 192 mM glycine buffer, pH 8.3, containing 20% methanol. The membranes were blocked for 1 h at room temperature using 1% bovine serum albumin (BSA) in PBS, pH 7.3, and then washed 6 times with PBS over 30 min. Incubation with the first antibody (neat NCRC-11 hybridoma tissue culture supernatant) was carried out for 3 h at room temperature and the membranes were washed as before. Horse radish peroxidase conjugated to $F(ab')_2$ fragments of sheep anti-mouse Ig (Amersham International, Amersham, Bucks.) at a dilution of 1/1000 in 1% BSA in PBS was applied to the nitrocellulose membranes and incubated at room temperature for 1 h. After washing 9 times with PBS over 45 min, peroxidase activity was detected by addition of 25 mg of 3,3′ diaminobenzidine tetrahydro-chloride (3,4,3′,4′-tetra-aminobiphenylhydrochloride – BDH, Poole, Dorset) in 50 ml 10 mM Tris-HCl, pH 7.4 with 50 $\mu$l 30% $H_2O_2$.

Figure 1 is an example of different phenotypes of urinary glycoproteins defined by the NCRC-11 antibody. The numbering system for the bands is as described by Karlsson *et al.* (1983) with reference to a standard panel of urines of known phenotype as defined by their reactivity with peanut lectin. Each of the four possible bands is present in one or more of these urine samples, with any individual displaying one or two bands (i.e. homozygote or heterozygote expression of gene products). In preliminary studies, it was demonstrated that the phenotype of any patient's high

**Figure 1** Immunoblotting of 5 concentrated urine samples. Samples were separated by SDS PAGE, electrophoretically transferred onto nitrocellulose paper and stained with the NCRC-11 antibody. Staining was visualized by incubation with peroxidase conjugated anti-mouse Ig followed by diaminobenzidine. Positions of molecular weight marker proteins (mol. wt $\times 10^{-3}$) and the top of the separating gel (open arrow) are shown to the left of the gel. The designations of the individual phenotypes according to Karlsson *et al.* (1983) are indicated to the right.

Correspondence: M.R. Price.
Received 11 December 1986; and in revised form 20 February 1987.

**Table I** Distributions of NCRC-11 antibody-defined phenotypes of urinary glycoproteins in breast cancer patients and normal individuals

*Breast cancer patients:*

Observed gene frequencies for NCRC-11 antibody defined urinary glycoproteins (bands 1 to 4):
f(1) = 0.34; f(2) = 0.38; f(3) = 0.21; f(4) = 0.07
Distribution of phenotypes:

| Phenotype | 1 | 2-1 | 2 | 3-1 | 3-2 | 3 | 4-1 | 4-2 | 4-3 | 4 | Total |
|---|---|---|---|---|---|---|---|---|---|---|---|
| Obs. no. | 12 | 17 | 21 | 15 | 11 | 5 | 7 | 3 | 4 | 0 | 95 |
| Exp. no.[a] | 11.0 | 24.5 | 13.7 | 13.6 | 15.2 | 4.2 | 4.5 | 5.1 | 2.8 | 0.5 | 95 |

$\chi^2 = 10.99$; $P > 0.05$

*Normal female control subjects:*

Observed gene frequencies for NCRC-11 antibody defined urinary glycoproteins (bands 1 to 4):
f(1) = 0.39; f(2) = 0.37; f(3) = 0.19; f(4) = 0.05
Distribution of phenotypes:

| Phenotype | 1 | 2-1 | 2 | 3-1 | 3-2 | 3 | 4-1 | 4-2 | 4-3 | 4 | Total |
|---|---|---|---|---|---|---|---|---|---|---|---|
| Obs. no. | 6 | 20 | 7 | 7 | 5 | 4 | 3 | 1 | 1 | 0 | 54 |
| Exp. no. | 8.2 | 15.6 | 7.4 | 8.0 | 7.6 | 2.0 | 2.1 | 2.0 | 1.0 | 0.1 | 54 |

$\chi^2 = 5.88$; $P > 0.05$

[a]The expected distribution was calculated from the observed gene frequencies assuming Hardy–Weinberg equilibrium.

molecular weight urinary glycoproteins was identical to that of glycoproteins produced by the tumour and found in the circulation – NCRC-11 defined antigens have not been detected at significant levels in the serum of normal individuals either by immunoblotting or by radioimmunoassay (Price *et al.*, 1987).

The phenotypes of NCRC-11 antibody-binding urinary glycoproteins of 95 breast cancer patients and 54 normal female control subjects were determined and the results are summarized in Table I which also includes the gene frequencies for the components in bands 1 to 4. The expected distribution of phenotypes was calculated from the observed gene frequencies assuming Hardy–Weinberg equilibrium. In both the breast cancer patients and normal individuals, the observed distribution did not deviate significantly from the calculated expected distributions (in accord with Hardy–Weinberg predictions for Mendelian co-dominant inheritance). Furthermore, differences in the distribution of phenotypes in the patient group in comparison to those of the normal controls did not reach statistical significance. Similarly, there was no significant ($P > 0.05$) preponderance of homozygote individuals in the patient group compared with the controls, as might be expected if diversity in NCRC-11 antigens confers any protective influence against neoplasia.

Clearly, for a more thorough investigation of the rarer phenotypes, it would be necessary to increase the number of samples analysed. However, it may be concluded from the present findings that the genetic polymorphism of high molecular weight glycoproteins identified using the NCRC-11 antibody plays no major role in the occurrence of breast cancer. Whether this system is of any influence in other epithelial malignancies with which NCRC-11 antigens are associated, or whether this antigen system influences tumour progression in any way, remains to be determined. The finding that the degree of staining of primary breast tumours with the NCRC-11 antibody is associated with good prognosis (Ellis *et al.*, 1984) still requires explanation, and the genetic polymorphism of the antigen involved is an additional factor to be considered in evaluating its prognostic capabilities.

These studies were supported by the Cancer Research Campaign. Thanks are expressed to Dr M. Williams (Professorial Surgical Unit, City Hospital, Nottingham) for making available the samples of breast cancer patients' urine used in this investigation.

### References

ASHALL, F., BRAMWELL, M.E. & HARRIS, H. (1982). A new marker for human cancer cells. 1. The Ca antigen and the Ca1 antibody. *Lancet*, **ii**, 1.

BRAMWELL, M.E., BHAVANANDAN, V.P., WISEMAN, G. & HARRIS, H. (1983). Structure and function of the Ca1 antigen. *Br. J. Cancer*, **48**, 177.

BURCHELL, J., DURBIN, H. & TAYLOR-PAPADIMITRIOU, J. (1983). Complexity of expression of antigenic determinants recognized by monoclonal antibodies HMFG-1 and HMFG-2, in normal and malignant human mammary epithelial cells. *J. Immunol.*, **131**, 508.

ELLIS, I.O., ROBINS, R.A., ELSTON, C.W. & 3 others (1984). A monoclonal antibody, NCRC-11, raised to human breast carcinoma. I. Production and immunohistological characterization. *Histopathology*, **8**, 501.

KARLSSON, S., SWALLOW, D.M., GRIFFITHS, R. & 4 others (1983). A genetic polymorphism of a human urinary mucin. *Ann. Hum. Genet.*, **47**, 263.

LAEMMLI, U.K. (1970). Cleavage of structural proteins during the assembly of the head of bacteriophage T4. *Nature*, **227**, 680.

PRICE, M.R., CROCKER, G., EDWARDS, S. & 6 others (1987). Identification of a monoclonal antibody defined breast carcinoma antigen in body fluids. *Eur. J. Cancer Clin. Oncol.* In press.

SHIMIZU, M. & YAMAUCHI, K. (1982). Isolation and characterization of mucin-like glycoprotein in human milk fat globule membrane. *J. Biochem.*, **91**, 515.

SWALLOW, D.M., GRIFFITHS, B., BRAMWELL, M., WISEMAN, G. & BURCHELL, J. (1986). Detection of the urinary 'PUM' polymorphism by the tumour binding monoclonal antibodies Ca1, Ca2, Ca3, HMFG-1 and HMFG-2. *Disease Markers*, **4**, 247.

TOWBIN, H., STAEHLIN, T. & GORDON, J. (1979). Electrophoretic transfer of proteins from polyacrylamide gels to nitrocellulose sheets: procedure and some applications. *Proc. Natl Acad. Sci. USA*, **76**, 4350.